# Clinical significance of plasmacytoid dendritic cells and myeloid-derived suppressor cells in melanoma

**DOI:** 10.1186/s12967-014-0376-x

**Published:** 2015-01-16

**Authors:** Ines Chevolet, Reinhart Speeckaert, Max Schreuer, Bart Neyns, Olga Krysko, Claus Bachert, Mireille Van Gele, Nanja van Geel, Lieve Brochez

**Affiliations:** Department of Dermatology, Ghent University Hospital, De Pintelaan 185, 9000 Ghent, Belgium; Department of Medical Oncology, UZ-Brussel, Brussels, Belgium; Department of Medical Oncology, Ghent University Hospital, Ghent, Belgium; Upper Airways Research Laboratory, Ghent University Hospital, Ghent, Belgium

**Keywords:** Melanoma, Plasmacytoid dendritic cell (pDC), Myeloid-derived suppressor cell (MDSC), Myeloid differentiation, Prognosis, Immunoprofiling

## Abstract

**Background:**

Immune markers in the peripheral blood of melanoma patients could provide prognostic information. However, there is currently no consensus on which circulating cell types have more clinical impact. We therefore evaluated myeloid-derived suppressor cells (MDSC), dendritic cells (DC), cytotoxic T-cells and regulatory T-cells (Treg) in a series of blood samples of melanoma patients in different stages of disease.

**Methods:**

Flow cytometry was performed on peripheral blood mononuclear cells of 69 stage I to IV melanoma patients with a median follow-up of 39 months after diagnosis to measure the percentage of monocytic MDSCs (mMDSCs), polymorphonuclear MDSCs (pmnMDSCs), myeloid DCs (mDCs), plasmacytoid DCs (pDCs), cytotoxic T-cells and Tregs. We also assessed the expression of PD-L1 and CTLA-4 in cytotoxic T-cells and Tregs respectively. The impact of cell frequencies on prognosis was tested with multivariate Cox regression modelling.

**Results:**

Circulating pDC levels were decreased in patients with advanced (P = 0.001) or active (P = 0.002) disease. Low pDC levels conferred an independent negative impact on overall (P = 0.025) and progression-free survival (P = 0.036). Even before relapse, a decrease in pDC levels was observed (P = 0.002, correlation coefficient 0.898). High levels of circulating MDSCs (>4.13%) have an independent negative prognostic impact on OS (P = 0.012). MDSC levels were associated with decreased CD3+ (P < 0.001) and CD3 + CD8+ (P = 0.017) T-cell levels. Conversely, patients with high MDSC levels had more PD-L1+ T-cells (P = 0.033) and more CTLA-4 expression by Tregs (P = 0.003). pDCs and MDSCs were inversely correlated (P = 0.004). The impact of pDC levels on prognosis and prediction of the presence of systemic disease was stronger than that of MDSC levels.

**Conclusion:**

We demonstrated that circulating pDC and MDSC levels are inversely correlated but have an independent prognostic value in melanoma patients. These cell types represent a single immunologic system and should be evaluated together. Both are key players in the immunological climate in melanoma patients, as they are correlated with circulating cytotoxic and regulatory T-cells. Circulating pDC and MDSC levels should be considered in future immunoprofiling efforts as they could impact disease management.

## Background

Melanoma is a highly immunogenic tumour that is capable of successfully evading the patients’ immune response. Evidence for an anti-tumoral immune reaction, as well as concomitant immunosuppressive mechanisms, can already be observed in the primary tumour and in tumour-free sentinel lymph nodes [1,2]. The possibility to integrate immune markers in the existing TNM-classification is currently being investigated in melanoma. The primary objective is to increase prognostic accuracy but it could also become a strategy to pre-select patients for adjuvant therapies [3]. Immunoprofiling initiatives such as the “Immunoscore” focus on markers in the primary and metastatic tumour site, mainly assessed by immunohistochemistry [4]. However, options to evaluate the immune status in a tumour-free patient during clinical follow-up are currently lacking. In this context, circulating biomarkers could be a practical approach.

In melanoma, it is at present unclear which circulating immune cell types confer the most powerful prognostic information. Besides T-cells, myeloid-derived suppressor cells (MDSCs) and dendritic cells (DCs) are the most elaborately studied circulating cell types, but research is often focused on the tumour microenvironment.

MDSCs are HLA-DR- lineage- CD33+ CD11b + cells that do not constitute a defined subset of cells but rather a group of phenotypically heterogeneous myeloid cells that have a common biological activity [5]. Two clinically relevant subsets have been defined, monocytic (CD14+) and polymorphonuclear (CD14-CD15+) MDSCs (resp. mMDSCs and pmnMDSCs). An expanding body of evidence shows increased levels of MDSCs in almost all cancer types, correlating with advanced clinical cancer stage and a worse prognosis [6,7]. Myeloid differentiation is often disturbed in cancer patients, leading to an accumulation of immunosuppressive immature myeloid cells such as MDSCs and reduced frequencies of mature, immunostimulatory dendritic cells (DCs) [8,9]. Tumour-derived factors are thought to inhibit the natural differentiation of immature myeloid cells, resulting in the accumulation of MDSCs [5]. This concomitant increase in MDSCs and decrease in mature DCs in the peripheral blood has been described in several cancer types [10]. High MDSC frequencies in the peripheral blood of melanoma patients have also been reported to have a negative impact on prognosis, but their relation to dendritic cells or lymphocytes is not well documented.

DCs are potent antigen-presenting cells that play a central role in developing anti-tumour immune responses. Two subsets of DCs have been defined in the blood and in lymphoid tissues, myeloid (CD11c+) and plasmacytoid (CD123+ CD11c-) DCs (resp. mDCs and pDCs) [9,11]. Additional surface markers for blood DCs exist; BDCA-1 and −3 define two distinct subsets of mDCs and BDCA-2 and BDCA-4 are present on pDCs. Many other surface markers further characterize these cells, as recently reviewed elsewhere [11,12]. The differentiation capacity of DCs is diminished in many cancers, resulting in lower frequencies of circulating mature DCs in patients with higher tumour stages or active disease [9,13,14]. In melanoma, circulating DC frequencies have been reported to be unchanged in stage I-III patients [13,14], and reduced in stage IV [14-16]. Similar patterns in DC alterations have been described in breast, liver, head and neck and lung cancer [10,17-19]. However, data on circulating DCs frequencies in untreated melanoma patients are limited and their prognostic relevance is unknown. The in vivo clinical relevance of these circulating subsets in melanoma therefore remains subject to debate [8].

Despite evidence that lymphoid cell types such as cytotoxic and regulatory T-cells are important in melanoma and are modulated by current immunotherapeutic strategies [20], many recent studies on circulating cell types in melanoma have focused on MDSCs alone and have left their relation to lymphoid cell types largely unexplored. The presence of regulatory T-cells (Tregs) in the melanoma tumour microenvironment confers a negative prognosis [21]. However, the prognostic relevance of circulating Tregs in untreated melanoma patients is unclear. Cytotoxic T-cells are powerful allies in the anti-tumoral immune response and their presence in the melanoma micro-environment is protective [1], but data on circulating cell frequencies in untreated patients are scarce.

Even in stage I and II melanoma a shift in systemic immune activity can be present and these systemic immune alterations have been reported to increase as patients develop metastatic disease. Many different cell types have separately been described in this context, but there is no consensus on which alterations have the predominant immunosuppressive effect. We therefore performed a comparative evaluation of the presence of different circulating immune subsets in untreated melanoma patients in different stages of disease, with a focus on DCs and MDSCs. The clinical relevance of circulating DC and MDSC subsets and their relation with regulatory and cytotoxic T-cells was assessed.

## Methods

### Patients

Sixty-nine patients with melanoma were included in this study, with a median follow-up time of 39 months after diagnosis and of 15 months after inclusion (inclusion was defined as the time of sample procurement). Venous blood samples were drawn during clinical follow-up, with a median interval of 21 months after diagnosis. Disease staging was done according to the 2009 American Joint Committee on Cancer system (AJCC). Local disease was defined as AJCC stage I and II, regional disease as AJCC stage III and systemic disease as AJCC stage IV. The local medical ethical committee approved this study; all included patients gave written informed consent. Detailed patient characteristics can be found in Table 1.

**Table 1:** **Patient characteristics**

Number of patients, n	69
Follow-up time since inclusion, months (median - IQR)	15 (6–35)
Follow-up time since diagnosis, months (median - IQR)	39 (20.5–108)
Age at diagnosis, years (median - IQR)	53 (41.5 – 60)
Female sex, % (n)	53.6 (37/69)
**Stage at inclusion, % (n)**	
Local (AJCC stage I & II)	46.4 (32/69)
Regional (AJCC stage III)	37.7 (26/69)
Systemic (AJCC stage IV)	15.9 (11/69)
Active disease at inclusion	20.3 (14/69) (3 stage III, 11 stage IV)
**Melanoma characteristics**	
Breslow (median, IQR)	1.60 (1.08 – 2.60)
Ulceration, % (n)	35.7 (20/56)
Sentinel invasion, % (n)	27.1 (16/59)
**Location of primary melanoma**	
Head & neck, % (n)	10.6 (7/69)
Trunk, % (n)	39.4 (26/69)
Extremities, % (n)	50 (33/69)
Unknown primary	3 (3/69)

IQR, interquartile range; AJCC, American joint committee on cancer.

### PBMC isolation

Peripheral blood mononuclear cells (PBMC) were isolated from heparinized venous blood by centrifugation on a Ficoll-Hypaque gradient (GE Healthcare, Uppsala, Sweden) within 4 h of venepuncture. The PBMCs were cryopreserved in liquid nitrogen in heat-inactivated foetal bovine serum (FBS) supplemented with 10% dimethyl sulphoxide (DMSO) until analysis. Cells were thawed by submersion at 37° for 1–2 minutes and resuspended in a medium containing Iscove’s Modified Dulbecco’s Medium (IMDM) supplemented with 20% FBS and 1% glutamine.

### Flow cytometry

MDSCs were characterized by the HLA-DR- lineage- (CD3, CD19, CD56) CD33+ CD11b + phenotype, mMDSCs are CD14+, pmnMDSCs are CD14-. Dendritic cells were characterized by the HLA-DR+ lineage- (CD3, CD14, CD16, CD19, CD20, and CD56) phenotype, pDCs are CD123+ CD11c- and mDCs are CD123- CD11c+. Tregs were defined as CD3+ CD4+ CD25+ FoxP3+ and cytotoxic T-cells as CD3+ CD8+ cells. All antibodies used in this study were fluorescently conjugated mouse anti-human monoclonal antibodies. The following antibodies were purchased from BD Biosciences; CD3 BV421 (563797), CD4 APC-Cy7 (561839), CD25 FITC (560990), CD33 BV421 (562854), CD11b APC-Cy7 (560914), CD123 BV421 (562517). The following antibodies were purchased from eBioscience; B7-H1 (PD-L1) PE-Cy7 (25-5983-42), CD8 APC (9017-0087-025), CD3 FITC (11-0038-41), CD19 FITC (11-0199-41), CD56 FITC (11-0569-41), CD14 APC (17-0149-41), CD11c APC (17-0116-41), HLA-DR PerCP-Cy5.5 (45-9956-42). For intracellular stainings, after surface staining PBMCs were fixed and permeabilized using Live/dead® fixable aqua dead cell stain (BD Biosciences), and then stained with antihuman CTLA-4 APC (BD Biosciences, 560938) and FoxP3 PerCP-Cy5.5 (eBioscience, 45-4776-42) antibodies. Live/dead staining was performed using an aqua Dead Cell Stain kit (Life Technologies Europe, Ghent, Belgium). Patients with less than 75% living cells were excluded (n = 4). Cells were analyzed on a FACS Canto™ II flow cytometer (BD Bioscience, Erembodegem, Belgium) using FlowJo software (Tree Star Inc, Ashland, OR, USA). For setting the gates, isotype and fluorescence-minus-one (FMO) controls were used. To provide a representative sample a median amount of 500000 cells were analysed (min 261000 – max 569750). Absolute cell counts were corrected for the number of acquired events during flow cytometry. Samples with less than 100000 cells were excluded (n = 1).

### Statistical analysis

Median values between 2 groups were compared by the Mann–Whitney *U*-test, between >2 groups with Kruskall-Wallis testing. To compare proportions of categorical variables, the Pearson’s Chi^2^ test or Fisher’s Exact test were used. To evaluate correlations, Spearman correlation coefficients (CC) were calculated. To assess prognostic relevance of continuous variables, ROC curve analysis was used to dichotomize them with the aid of the online tool “cut-off finder” [22]. All statistical analyses were performed using SPSS 21.0 (SPSS Inc, Chicago, IL, USA), a P-value (double-sided) less than 0.05 was considered statistically significant.

## Results

Flow cytometry was performed to quantify MDSC and DC subsets, Tregs and cytotoxic T-cells in PBMCs from melanoma patients (Table 1). Table 2 summarizes the mean detected cell frequencies for all cell types, and the cut-off points for the dichotomized cell frequencies that were used in Cox regression models. As a first step, all immune subsets were compared for their relevance with regard to clinical variables such as disease stage and outcome. This showed us that both pDCs and MDSCs are associated with disease stage and have an impact on prognosis. Therefore further analyses were focused on these cell types, as outlined below.

**Table 2:** **Frequencies of circulating immune subsets in melanoma patients**

**Cell type**	Cell frequency (mean % - SD)*	Dichotomization cut-off (%)*	**Patients with “high” cell levels (%)**
**Dendritic cells (DC)**	1.54 (0.54)		
Plasmacytoid DCs	0.34 (0.18)	0.2515	66.7
Myeloid DCs	0.96 (0.40)		
**Myeloid-derived suppressor cells (MDSC)**	4.00 (2.29)	4.13	33.3
Monocytic MDSC	2.94 (2.81)		
Polymorphonuclear MDSC	1.04 (0.64)		
**CD3+ cells**	43.60 (9.32)	40.25	68.2
Cytotoxic T-cell	14.79 (6.06)		
Regulatory T-cell	4.77 (1.35)		
CTLA-4 expression by Tregs	92.32 (4.01)	93.7	37.3

DC, dendritic cell; MDSC, myeloid-derived suppressor cell; Treg, regulatory T-cell; SD, standard deviation.

* Percentage of live cells, except for Tregs (percentage of CD4+ cells).

### Myeloid-derived suppressor cells (MDSCs)

**Figure 1:**
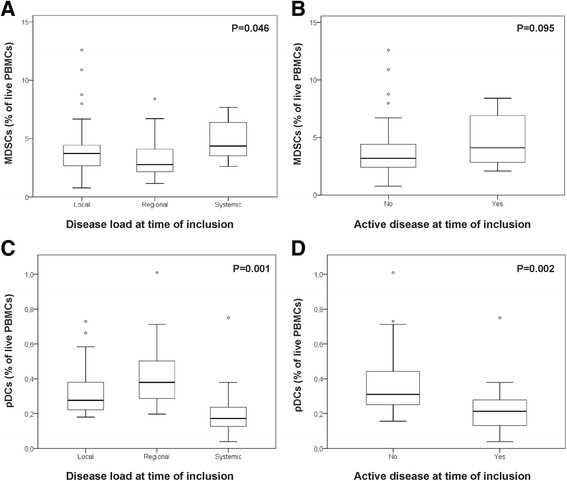
**pDC and MDSC frequencies according to melanoma stage and activity.** Box-and-whisker plots showing variations in the levels of circulating myeloid-derived suppressor cells (MDSCs; **A** and **B**) and plasmacytoid dendritic cells (pDCs; **C** and **D**) according to the presence of systemic or active disease at the time of inclusion. Circulating cell frequencies are expressed as a percentage of live peripheral blood mononuclear cells (PBMCs).

Melanoma patients with systemic disease have significantly higher frequencies of circulating MDSC (P = 0.046). There was a trend towards higher MDSC frequencies in patients with active disease at time of inclusion, but this did not reach significance (Figure 1).

**Figure 2:**
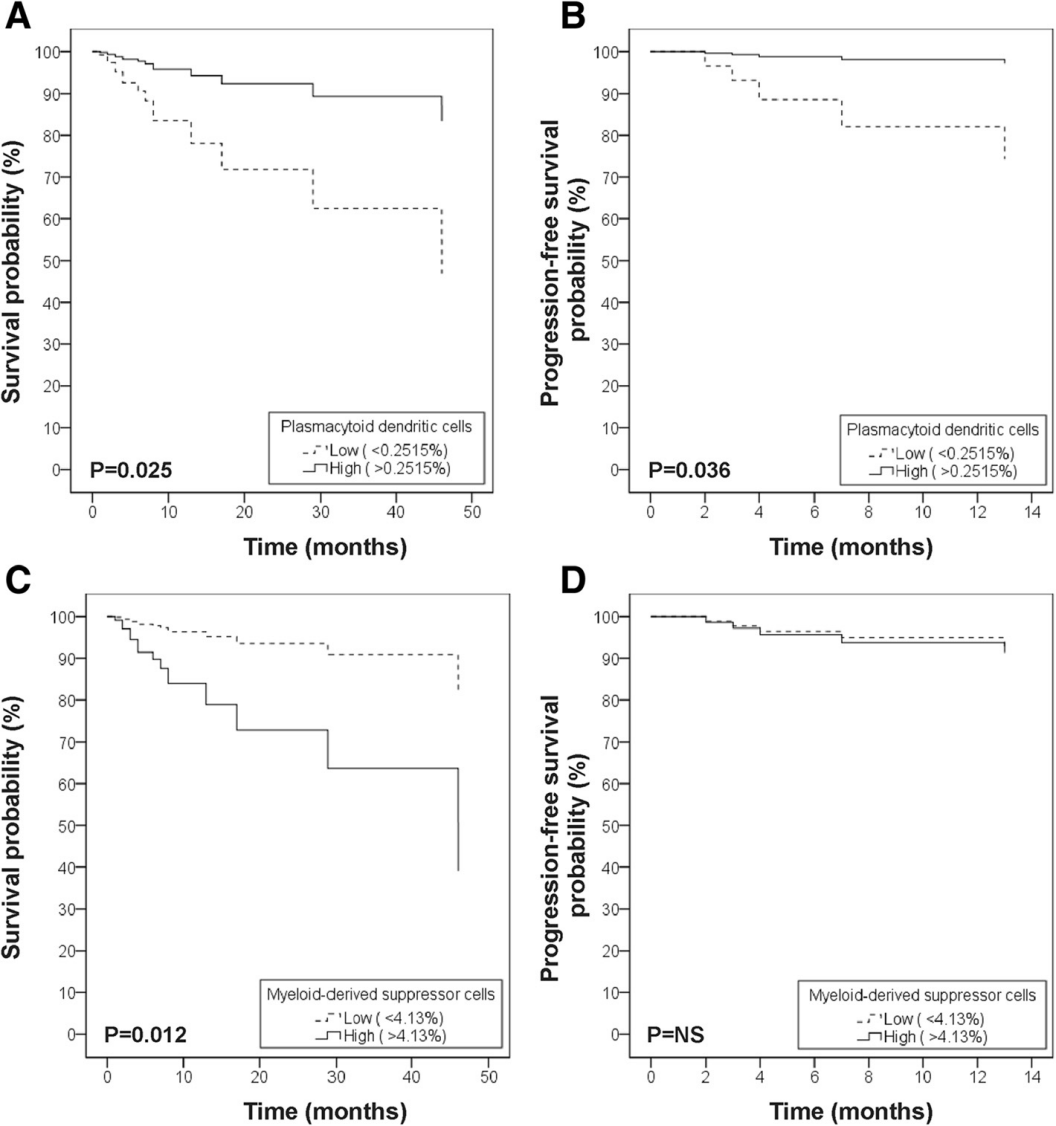
**Impact of pDC and MDSC frequency on overall and progression-free survival.** Cox regression analysis of overall **(A and C)** and progression-free **(B and D)** survival according to the levels of circulating plasmaytoid dendritic cells **(A and B)** or myeloid-derived suppressor cells **(C and D)**, after adjustment for disease stage. Time is defined as follow-up time since blood sample procurement. Percentages should be interpreted as percentages of live peripheral blood mononuclear cells (PBMCs).

High levels of circulating MDSCs (>4.13%) conferred a negative impact on OS (Log Rank test, P = 0.002). This effect was independent of disease stage (Figure 2, P = 0.012, HR 4.77, CI 1.42-16.04). There was no impact of MDSC frequency on progression-free survival (PFS, Figure 2). Patients who died of melanoma after inclusion showed a trend towards more circulating MDSCs at the time of inclusion (P = 0.073).

### The relation of MDSCs with the systemic immune context

To further evaluate how MDSCs are related to systemic immunity, their relation to cytotoxic T-cells and Tregs was also assessed (Table 3).

**Table 3:** **Association of myeloid-derived suppressor cells (MDSCs) and plasmacytoid dendritic cells (pDCs) with melanoma disease course and circulating immune markers**

	**MDSCs**	**pDCs**
**Melanoma activity and prognosis**		
Systemic disease	Increased (P = 0.046)	Decreased (P = 0.001)
Active disease	Increased (P = 0.095)	Decreased (P = 0.002)
Death	Increased (P = 0.073)	Decreased (P = 0.009)
**Other immune markers**		
CD3 cells	Inverse (P < 0.001)	None
CD8 cells	Inverse (P = 0.017)	None
PD-L1+ CD8 cells	Positive (P = 0.033)	Inverse (P = 0.044)
Regulatory T-cells	Inverse (P = 0.007)	None
CTLA-4 expression in Tregs	Positive (P = 0.003)	None

PD-L1, Programmed-Death Ligand 1; CTLA-4, Cytotoxic T Lymphocyte-Associated Antigen 4; Treg, regulatory T-cell.

The frequency of CD3+ cells was inversely correlated with MDSCs (P < 0.001, CC −0.519), mMDSCs (P < 0.001, CC −0.454) and pmnMDSCs (P = 0.004, CC −0.349). The cytotoxic T-cell frequency was also inversely correlated with MDSCs (P = 0.017, CC −0.294). On the other hand, there was a positive correlation between PD-L1+ cytotoxic T-cells and MDSCs (P = 0.033, CC 0.263), mainly observed in pmnMDSCs (P = 0.008, CC 0.323). These data suggest that high MDSC frequencies are associated with relative lymphopenia and with an immune climate that is unfavorable for cytotoxic T-cells.

The percentage of MDSCs was inversely correlated with the percentage of Tregs (P = 0.007, CC −0.327). However, the proportion of highly CTLA-4-positive Tregs was positively correlated with the percentage of MDSCs (P = 0.002, CC 0.365) and mMDSCs (P = 0.008, CC 0.321). This was also the case for the mean fluorescence intensity (MFI) of CTLA-4 and MDSCs (P = 0.029, CC 0.266). These data suggest that high MDSC frequencies are associated with a higher suppressive capacity of circulating Tregs, but not with higher Treg frequencies.

Patients who died of melanoma during follow-up had a trend towards more circulating highly CTLA-4+ Tregs (P = 0.081). A high percentage of CTLA-4 positivity in circulating Tregs (>93.7%) conferred a negative prognosis (Log Rank test, P = 0.003), independent of disease stage (P = 0.040, HR 3.80, CI 1.06-13.70).

### Plasmacytoid dendritic cells (pDCs)

Melanoma patients with systemic disease have significantly lower frequencies of circulating pDCs (P = 0.001). This decrease in pDCs was also seen in patients with active disease at the time of inclusion (P = 0.002) (Figure 1). In patients who were disease-free at the time of inclusion but who presented with disease relapse or progression in the months after inclusion, a decline in pDCs frequency could already be seen. The shorter the time frame between inclusion and relapse, the lower the observed frequency of pDCs (P = 0.002, CC 0.898).

A low amount of circulating pDCs (<0.2515%) had a negative prognostic impact on OS, independent of disease stage (Figure 2; P = 0.025, HR 4.17, CI 1.20-14.52). In patients who were disease-free at the time of inclusion, low frequencies of circulating pDCs were also associated with a shorter PFS, independent of disease stage (Figure 2; P = 0.036, HR 10.29, CI 1.162-90.90).

### The relation of pDCs with the systemic immune context

To further evaluate the impact of pDCs on systemic immunity, their relation to Tregs and cytotoxic Tcells was also assessed by flow cytometry. An inverse correlation between pDC frequency and the percentage of PD-L1+ cytotoxic T-cells was found (P = 0.044, CC −0.249). No correlation with Treg frequency or CTLA-4 expression was detected.

### Clinical relevance of MDSCs compared to pDCs

There was no inverse relation between the frequency of DCs and MDSCs in general. However, when evaluating the relationship between MDSC and DC subtypes, a significant inverse correlation was found between pDCs and MDSCs (P = 0.004, CC −0.338). This was due to an increase in the absolute number of mMDSCs (P = 0.010, CC −0.307), not pmnMDSCs. The observed changes in pDC and MDSC frequencies in patients with systemic disease were also reflected by an alteration of the MDSC/DC ratio (P = 0.048). These data suggest that low pDC frequencies are associated with a defective maturation of myeloid cells, and an accumulation of MDSCs.

To assess the relative importance of pDCs and MDSCs with regard to prognosis (impact on OS), a combined Cox regression model was made (Table 4, upper part). MDSC and pDC frequencies had a similar impact on OS, independent of each other and of disease stage. When CTLA-4+ Treg levels or CD3+ T-cell frequencies were separately added to this model, both lost their effect on prognosis, whereas pDCs did not (P = 0.008 for adding CTLA-4, P = 0.019 for adding CD3). These results indicate that circulating myeloid cells (principally pDCs) have a superior impact on prognosis (OS) compared to T-cells.

**Table 4:** **Combined Cox (upper part) and logistic (lower part) regression models comparing circulating myeloid-derived suppressor cells and plasmacytoid dendritic cells in melanoma patients**

**Combined Cox regression model: pDC vs MDSC & overall survival**					
	**Coefficient**	**P-value**	**HR**	**95% Confidence Interval for HR**	
				**Lower**	**Upper**
Stage at inclusion	1.87	<0.001	6.46	2.39	17.46
MDSC frequency	−1.61	0.009	4.98	1.50	16.67
pDC frequency	1.49	0.019	4.42	1.27	15.33
**Logistic regression model: pDC and MDSC & systemic disease**					
	**Coefficient**	**P-Value**	**OR**	**95% Confidence Interval for OR**	
				**Lower**	**Upper**
Stage at diagnosis	−0.702	0.407	0.496	0.095	2.601
MDSC frequency	−0.248	0.753	0.78	0.167	3.655
pDC frequency	1.741	0.032	5.705	1.159	28.091

pDC, plasmacytoid dendritic cell; MDSC, myeloid-derived suppressor cell; HR, hazard ratio; OR, odds ratio.

As pDCs and MDSCs emerge as the most prognostically relevant cell types, we next assessed their relative importance to each other in predicting the presence of systemic disease. As indicated in Table 4 (lower part), a logistic regression model was made. A low pDC frequency at the moment of inclusion could predict the presence of systemic disease, independent of the patients’ stage at the time of inclusion and independent of MDSC frequency. MDSC frequency itself had no significant predictive quality.

## Discussion

Various systemic immune alterations have been described in melanoma patients, but a direct comparison of their clinical relevance is often lacking. In this study, we demonstrated that an increased amount of circulating MDSCs and a decreased number of pDCs were associated with systemic disease and had both independent negative prognostic value, although they were inversely correlated. Both MDSCs and pDCs were linked with T-cell anergy. Nonetheless, pDCs and MDSCs were the most prognostically relevant cell types.

In this patient cohort, elevated MDSC frequencies were seen in stage IV melanoma patients, which is in accordance with previous publications [23-25]. The accumulation of MDSCs in melanoma is most prominent in stage IV patients, but has been reported as early as stage I [26]. Most authors agree that MDSCs are upregulated in melanoma patients compared to healthy controls [23,25,26]. Whether pmnMDSCs or mMDSCs dominate depends on the cancer type. Based on literature and the observations made in this patient set, mMDSCs appear to have higher impact in melanoma [27,28]. A negative impact of high CD14- and CD14+ MDSCs on OS and PFS was previously described in stage IV melanoma patients [25,29]. Significant increases in circulating MDSC frequencies have also been detected in the blood of patients with a.o. glioblastoma, breast, colon, lung and kidney cancer [5,30].

We observed significantly lower circulating pDC frequencies in stage IV melanoma patients. The changes in total DC frequencies in advanced melanoma have previously been attributed to pDCs rather than mDCs [14,15]. Accordingly, we did not observe significant changes in mDC frequencies, contrary to reduced pDC levels in stage IV patients. Remarkably, we observed that a decline in pDC levels had a negative prognostic effect on both OS and PFS, independent of disease stage or other cell frequencies. Previous studies have reported a negative prognostic effect of the presence of pDCs within the tumour site [31,32], but to our knowledge this is the first study to report an independent negative prognostic effect of low circulating pDC frequencies in untreated melanoma patients. Moreover, low pDC frequencies were not only associated with a decreased PFS, but were also already reduced up to a year before relapse was clinically diagnosed, suggesting that low pDC frequencies could have a predictive value in disease-free patients. A possible explanation is that pDC frequencies are regulated by the tumour itself and that this regulation takes place very early in the metastatic process. Alternatively, the observed low pDC frequencies could also be an indicator of a failing anti-tumoral immune response, making the patient more susceptible to relapse. The relevance of pDC frequencies in detecting early disease progression should be further investigated in prospective trials with longitudinal follow-up samples.

Our data demonstrate a disturbed myeloid differentiation in melanoma, resulting in an accumulation of MDSCs and a decline in pDCs. To our knowledge, there are no studies directly reporting this effect in melanoma patients yet. The independent prognostic effect of both pDCs and MDSCs suggests that even a partial dysfunction of myeloid differentiation is sufficient to negatively influence melanoma disease course. In our dataset, the effect of pDCs on OS and PFS outweighs that of MDSCs (Table 4). An alternative interpretation could be that MDSC frequency is related to the actual tumour load, whereas adequate pDCs levels could also have a protective effect on disease progression. Regardless of which myeloid cell type dominates over the other during the course of the disease, it is clear that the myeloid lineage is globally altered in melanoma as a single system involving both differentiated myeloid cells and their pathologically activated immature progenitors [30]. Additionally, our combined Cox regression model showed that the prognostic importance of circulating myeloid cells dominates over circulating cytotoxic T-cells and to a lesser extent regulatory T-cells, as the latter two lose their impact on prognosis when adjusting for pDC or MDSC frequencies.

Over the last few years, immunoprofiling efforts are increasing as immunotherapeutic strategies are gaining importance in the management of melanoma and other cancers. Several (combinations of) markers with predictive or prognostic quality have been suggested, mainly focusing on the tumour microenvironment [4,33]. Based on our data, we conclude that circulating pDCs and MDSCs should be considered in future prognostic profiling studies. One limitation of our study in this respect is the use of cryopreserved PBMC samples, which impedes a direct comparison with studies using fresh whole blood samples. MDSCs have already been investigated as possible predictive biomarkers for immunotherapy in melanoma and other malignancies. Low mMDSC levels were associated with clinical response and improved OS in melanoma patients treated with ipilimumab [34,35]. Moreover, mMDSC levels have been reported to be inversely correlated with the presence of tumour-specific T-cells and with a CD8+ T-cell rise on ipilimumab therapy [29,36]. The inverse correlation between mMDSC and (antigen-specific) cytotoxic T-cell levels that has been observed in independent studies including ours [37], also raises the question whether mMDSC-related immune suppression could be limiting the therapeutic benefit of ipilimumab. Alternatively, high mMDSC levels could also be a marker for deficient myeloid cell maturation leading to a pDC deficit that hampers efficient immune activation after ipilimumab therapy.

Recently, Schilling and colleagues reported that mMDSC frequencies decline in patients who have a response to vemurafenib and rise again when progressive disease occurs. The inhibitory effect of vemurafenib on mMDSCs was present in vitro as well as in vivo [38]. Finkelstein and colleagues reported that high DC/MDSC ratios and low pretreatment MDSC levels could predict response to high-dose IL-2 therapy in patients with melanoma and renal cell carcinoma [39]. In our patient cohort, pDC frequencies had the highest impact on prognosis. Furthermore a gradual decline in pDCs levels seemed to occur before relapse, but as this was a cross-sectional study these data need to be confirmed in a longitudinal study with multiple follow-up samples during disease course. However, our data do suggest that pDCs might also be valuable candidates in predictive immune profiles. The prominent prognostic role of circulating pDCs also warrants further research into possible therapeutic strategies, for example with Toll-like receptor stimulating drugs which have been shown to enhance pDC activation in the skin [40].

## Conclusions

Plasmacytoid dendritic cells (pDC) and myeloid-derived suppressor cells (MDSC) in the peripheral blood should be regarded as one system and thus be evaluated together. Both are key players in the immunological climate in melanoma patients, as they are correlated with circulating cytotoxic and regulatory T-cells. The independent prognostic impact of pDCs and MDSCs makes these cell types attractive for future prognostic and possibly even predictive immunoprofiling efforts.

## Abbreviations

AJCC: American Joint Committee on Cancer system
CTLA-4: Cytotoxic T Lymphocyte-Associated Antigen 4
DC: Dendritic cells
mDC: Myeloid DC
MDSC: Myeloid-derived suppressor cells
mMDSC: Monocytic MDSC
OS: Overall survival
PBMC: Peripheral blood mononuclear cells
pDC: Plasmacytoid DC
PD-L1: Programmed-Death Ligand 1
PFS: Progression-free survival
pmnMDSC: Polymorphonuclear MDSC
Treg: Regulatory T-cell
